# CD109 and squamous cell carcinoma

**DOI:** 10.1186/s12967-018-1461-3

**Published:** 2018-04-06

**Authors:** Ruixia Qi, Fengyun Dong, Qiang Liu, Yoshiki Murakumo, Ju Liu

**Affiliations:** 1 Taishan Medical College, Tai’an, Shandong China; 2 Laboratory of Microvascular Medicine, Medical Research Center, Shandong Provincial Qianfoshan Hospital, Shandong University, 16766 Jingshi Road, Jinan, 250014 Shandong China; 3 Department of Pathology, Kitasato University School of Medicine, Sagamihara, Japan

**Keywords:** CD109, Squamous cell carcinoma (SCC), TGF-β signaling pathway, STAT3

## Abstract

Squamous cell carcinoma (SCC) is well-known for its high rate of metastasis with poor prognosis. CD109 is a glycosylphosphatidylinositol-anchored cell-surface glycoprotein. Recently, CD109 emerges as a potential biomarker and a therapeutic target for SCCs. Accumulating studies have reported that CD109 is highly expressed in human SCCs of multiple organs, and may contribute to the progression of SCCs. In this review, we summarized the findings on expression pattern of CD109 in SCCs, and discussed the molecular mechanisms underlying the roles of CD109 in pathogenesis of SCCs.

## Background

Squamous cell carcinoma (SCC), also known as prickle cell carcinoma, is one of the leading causes of cancer-related death in worldwide. SCC is a malignant epithelial tumor [1], arising in tissues that provide a barrier between an organism and the environment, such as the skin, oral, cavity, esophagus and lung [2]. At an earlier stage, SCC is accompanied by epidermal keratinization and ulcer formation in the mucosal surface, and the deeper tissues are invaded by SCCs at a later stage. For microscopic appearance, the SCC cells arranged in nests, which are surrounded of polygonal cells with distinct cell borders and hyperchromatic nuclei. SCCs have a high tendency to metastasize, usually through regional lymph nodes, and might cause systematic damages of multiple organs. Surgery, laser therapy, and radiation continue to be the most broadly used treatment for SCCs [1]. The unique cytomorphologic features of SCC variants lead to distinct treatment and outcomes [1].

**Fig. 1:**
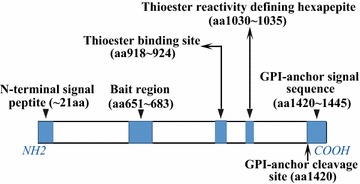
The structure of CD109 protein. The full-length of CD109 protein comprises 1445 amino acid (aa), including a 21 aa N-terminal signal peptide, a putative bait region (aa 651–683), a thioester binding site (aa 918–924), a thioester reactivity defining hexapeptide (aa1030–1035) and a C-terminal consensus GPI-anchor signal sequence (aa 1420–1445) with the cleavage predicted to occur after amino acid 1420

Cell surface antigen CD109 is a glycosylphosphatidylinositol (GPI)-linked glycoprotein of approximately 170 kDa and a member of the a2 macroglobulin (a2M)/C3, C4, C5 family of thioester-containing proteins [3]. The human *CD109* gene is located in chromosome 6q, constituting approximately 3.3% of the total CD109 genomic sequence with its 33 exons [4]. The isolated CD109 cDNA comprises a 4335 bp open-reading frame encoding a 1445 amino acid (aa) [3]. The CD109 protein contains a 21 aa N-terminal leader peptide, a putative bait region (aa 651–683), a thioester binding site (aa 918–924), a thioester reactivity defining hexapeptide (aa1030–1035) and a C-terminal consensus GPI-anchor signal sequence with the cleavage predicted to occur after amino acid 1420 (Fig. 1) [5]. The N-terminal leader peptide anchor the protein to the inner membrane within the periplasm [6] and the N-terminal fragment of CD109 secreted from cells after cleavage by the furin protease [7]. The protease cleavage of a largely disordered bait region activates CD109, resulting in a conformational change that traps the protease in a cage-like structure and exposes the highly reactive thioester bond. The thioester binding site which on activation can covalently link CD109 to lysine residues on the surface of the attacking protease. Besides, the C-terminal consensus GPI-anchor signal sequence is also known as the receptor-binding domain [6].

CD109 was first identified as a cell-surface antigen by a monoclonal antibody raised against the primitive lymphoid/myeloid cell line KG1a [8]. Previous studies reported that CD109 is expressed on a subset of fetal and adult CD34^+^ positive bone marrow mononuclear cells, activated T lymphoblasts, activated platelets, endothelial cells, mesenchymal stem cell subsets and several human tumor cell lines [9], but not expressed in resting human T cells, platelets or peripheral blood leukocytes [10]. In particular, CD109 has been detected in SCCs of several organs, including lung [11, 12], esophagus [11, 13], uterine cervix [14], skin [15], penis [16], oral cavity [17] and gallbladder [18]. CD109 is a component of the transforming growth factor-β1 (TGF-β1) receptor system and negatively regulates TGF-β1 signaling [5]. Loss and gain of function studies suggested that CD109 may be a molecular target for the development of new therapeutics for SCCs of various tissue origins [11–18]. In this review, we will present a summary of the current knowledge of CD109 and its relationship with SCCs.

### CD109 expression in SCCs

In tumor tissues, CD109 was immunohistochemically detected in SCCs [11–18] as well as urothelial carcinomas [19], malignant melanomas [20], basal-like breast carcinomas [21], myxofibrosarcoma [22], epithelial sarcomas [23] and glioma [24]. In particular, Shiraki et al. [24] reported CD109-positive perivascular tumor cells in human lower-grade glioma tissues and in a mouse model recapitulated human glioma, suggesting a key role of CD109 for this disease. Previous studies have shown that the high expression of CD109 in SCCs and the limited expression in normal squamous cells (Table 1) [17]. Furthermore, CD109 is highly expressed in well-differentiated SCCs rather than in moderately or poorly differentiated SCCs, thus the expression level of CD109 is inversely correlated with tumor grade [15, 17].

**Table 1:** Expression of CD109 in squamous cell carcinomas

Organ origin	Total no. of samples	No. of positive samples	Positive rate (%)	Inverse correlation with tumor grade	References
Lung	26	24	92.3	Not mentioned	[12]
Esophagus	79	78	98.7	Yes	[13]
Uterine cervix	10	10	100	Not mentioned	[14]
Cutaneous	20	18	90	Yes	[15]
Penis	41	41	100	Yes	[16]
Oral cavity	68	62	91.18	Yes	[17]
Gallbladder	15	13	86.7	Not mentioned	[18]

#### CD109 and lung SCC

Lung squamous cell carcinoma (LSCC) is one of the major subtypes of non-small-cell lung cancer (NSCLC), and accounts for approximately 20–30% of cases of NSCLCs [25]. CD109 expression was examined in human lung cell carcinomas by quantitative RT-PCR, which showed a significantly higher expression of CD109 in squamous cell carcinomas, but not in adenocarcinomas, large-cell carcinomas or small-cell carcinomas [11]. In addition, Sato et al. [12] used a CD109 antibody to analyze CD109 expression in normal lung and lung carcinoma tissues. In the normal lung tissues, CD109 expression was confined mainly to basal cells of the bronchial and bronchiolar epithelia [12]. In lung carcinoma tissues, strong immunoreactivity was preferentially detected in LSCCs but not in other types of lung carcinomas [12]. These findings indicate that CD109 is specifically up-regulated in LSCCs.

#### CD109 and esophagus SCC

Esophagus squamous cell carcinoma (ESCC) accounts for one-sixth of all cancer-related mortality, occurring at a higher incidence in Asian countries [26]. The prognosis of ESCC remains poor, and the 5-year survival rate of ESCC is about 14–22% [27]. Recent studies suggest that altered expression of TGF-β receptors contributes to ESCC progression, and elevated expression of inhibitory proteins of TGF-β signaling correlates with poor prognosis of ESCC [28, 29]. Hashimoto et al. [11] examined CD109 expression in ESCC tissues by quantitative RT-PCR, and CD109 mRNA transcription was upregulated in about 50% of the ESCCs. Later, the protein level of CD109 in ESCC was examined by immunohistochemistry on tissue microarrays (TMA) [13]. In the non-diseased esophageal tissue, CD109 expression was restricted in cytosol of the stratified epithelial cells at a weak level. In all the ESCC samples from the TMAs, strands of malignant squamous epithelial cells displayed strong CD109 staining in the cytosol. No CD109 positive staining was observed in other cell types. Furthermore, CD109 expression was higher in well- and moderately-differentiated ESCCs than the poorly differentiated ones [13].

#### CD109 and uterine cervix SCC

Cervical (uterine cervix) cancer accounts for about 12% of cancer-related death of women [30, 31]. SCCs account for 80–85% of all cervical cancers [32]. Zhang et al. [14] showed that CD109 expression was significantly higher in cervical SCCs than that in endometrial adenocarcinomas. They also investigated CD109 expression in five human cervical carcinoma cell lines, and observed high levels of CD109 expression in two SCC cell lines.

#### CD109 and cutaneous SCC

Cutaneous squamous cell carcinoma (CSCC) is the second most common type of non-melanoma skin cancer (NMSC) with a constantly increasing incidence [33]. Although CSCC have a generally favorable prognosis, there is still approximately 1.5–2% of patients die from this disease [34]. CD109 expression in CSCCs has been examined by immunohistochemistry on TMAs [15]. In the normal skins, CD109 was weakly expressed in the basal layer of epidermal cells, while strands of malignant squamous epidermal cells displayed strong CD109 staining. Besides, CD109 expression was inversely correlated with CSCC grades. Like ESCCs, the expression of CD109 was higher in well- and moderately-differentiated CSCCs than the poorly differentiated ones [15]. Penile squamous cell carcinoma (PSCC) is a subtype of CSCCs and has rarely been studied [35, 36]. Dong et al. demonstrated that CD109 protein is highly expressed in malignant squamous cells of PSCCs compared with normal penile tissues on TMAs. Furthermore, the expression pattern is validated on fresh surgical PSCC samples by immunofluorescence, qRT-PCR and western blotting, suggesting that CD109 may be a biomarker for PSCC [16].

#### CD109 and oral cavity SCC

Oral squamous cell carcinoma (OSCC) accounts for 2–3% of all cancers worldwide [37]. Loss of function mutation in TGF-β type II receptor is a frequent event for oral cavity SCC [2]. CD109 expression in normal oral tissues and OSCC tissues from 124 patients was examined by immunohistochemical staining. High levels of CD109 expression were frequently detected in SCCs and premalignant lesions of the oral cavity, but not in normal squamous epithelia [17]. Moreover, the expression level of CD109 was significantly higher in well-differentiated OSCCs than in moderately or poorly differentiated OSCCs, which implies that CD109 expression is correlated with the differentiation stages of OSCCs [17]. In addition, OSCC cell lines overexpressing CD109 exhibited accelerated cell growth in vitro [17], implicating that CD109 involves in the progression of OSCCs.

#### CD109 and gallbladder SCC

Gallbladder cancer (GBC) is the most aggressive of the biliary cancers with shortest median survival [38]. Gallbladder squamous cell carcinoma (GSCC) accounts 3% of the malignant neoplasm of this organ [39]. Subtypes of GBCs tissues including adenocarcinoma (AC), squamous cell carcinoma (SCC), and adenosquamous carcinoma (ASC) were examined on TMAs by immunohistochemical staining with a CD109 antibody. CD109 staining was negative in all normal gallbladder tissues and AC tissues. Meanwhile, CD109 positive cells were found in 86.7% of SCCs and 91.7% of ASCs. As CD109 is distinctly expressed in malignant sqamous cells in gallbladder, CD109 may be a diagnostic marker for gallbladder SCCs and ASCs [18].

### TGF-β signaling pathway and SCCs

The TGF-β signaling pathway is involved in many cellular processes including cell growth, cell differentiation apoptosis, and cellular homeostasis [40, 41]. The family of TGF-β ligands, TGF-β1, TGF-β2 and TGF-β3, binds to specific transmembrane type I and type II serine/threonine kinase receptors (TGF-βR1 and TGF-βR2) [42], resulting in activation of TGF-βR1 kinase activity [42, 43]. The activated TGF-βR1 then propagates the signal by phosphorylating its intracellular substrates, R-SMADs (Smad2 and Smad3) [44]. Smad2 and Smad3 interact with TGF-βR1 and SARA (Smad anchor for receptor activation), a FYVE domain protein that interacts directly with Smad2 and Smad3, SARA functions to recruit Smad2 to the TGF-β receptor [45], then the phosphorylated R-SMADs form heteromeric complexes with Co-SMAD (Smad4) [44]. After the phosphorylation and subsequent complexes with Smad4, these R-Smads complexes are released from TGF-βR1 and SARA [45], then translocate into the nucleus where they interact with transcription factors that recruit them to specific promoter elements of target genes [41, 44, 46].

Receptor endocytosis is a pivotal regulatory mechanism in signal transduction. TGF-β receptors are internalized via both clathrin- and caveolae-dependent pathways. Internalization of the TGF-β receptors via the clathrin-coated pits has been linked with signaling via Smad2/3 and receptor recycling. In contrast, TGF-β receptor localization in caveolae is associated with downregulation of Smad2/3 signaling and receptor degradation following ubiquitination by the E3-ubiquitin ligase Smurf2 [47]. However, inhibitory Smads (Smad6 and Smad7) form a distinct subclass of Smads that act in an opposing manner to R-Smads and antagonize signaling [48]. They may compete with R-Smads for binding to activated TGF-βR1 and thus to inhibit the phosphorylation of R-Smads [41]. In addition, they recruit E3-ubiquitin ligases to the activated TGF-βR1, resulting in receptor ubiquitination and degradation, and termination of signaling [44].

Dysregulation of the TGF-β pathway has been implicated in multiple types of cancer [49]. Studies have demonstrated that TGF-β signaling elicits a preventative effect during the earlier stages of tumorigenesis, but a suppressive effect during the later in tumor development [50]. Mutations in the *TGF*-*βR1* gene have also been found in SCCs of the skin, suggesting that the inactivation of TGF-β leads to the initiation of SCCs [2].

### CD109 and TGF-β signaling pathway

CD109 is a TGF-β co-receptor [11], and modulates TGF-β signaling receptor activity in a cell-specific manner [49, 51]. On the cell surface, CD109 negatively regulates the TGF-β1 signaling pathway via formation of a receptor complex with TGF-βR1 and TGF-βR2 in human keratinocytes [5]. TGF-β receptors degradate following ubiquitination by the E3-ubiquitin ligase Smurf2 [47], and are internalized via both clathrin-dependent and caveolae-dependent pathways [52]. Bizet et al. [47] demonstrated that CD109 associates with caveolin-1 and promotes TGF-β receptor endocytosis. In addition, CD109 promotes localization of the TGF-β receptors into the caveolar compartment in the presence of ligand and facilitates TGF-β receptor degradation. CD109 also regulates the localization and the association of Smad7/Smurf2 with TGF-βR1. The inhibitory effects of CD109 require Smad7 expression and Smurf2 ubiquitin ligase activity [49]. Furthermore, CD109 can be released from the cell surface by cellular lipases such as phosphatidylinositol-specific phospholipase C (PI-PLC). The soluble form of CD109 retains its ability to bind TGF-β1 and confiscate it away from the TGF-β receptors [53].

However, Vorstenbosch et al. [54] reported that CD109 differentially regulated TGF-β-induced ALK1-Smad1/5 versus ALK5-Smad2/3 pathways (ALK1 and ALK5 are all TGF-β type I receptors). They found that TGF-β signaling inhibits endothelial cell proliferation and migration, while TGF-β signaling also induces these processes via ALK1-Smad1/5 [54]. They demonstrate that ALK1 is expressed and co-localizes with CD109 in mouse keratinocytes and that mice overexpressing CD109 in the epidermis display enhanced ALK1-Smad1/5 signaling, but decreased ALK5-Smad2/3 signaling [54].

**Fig. 2:**
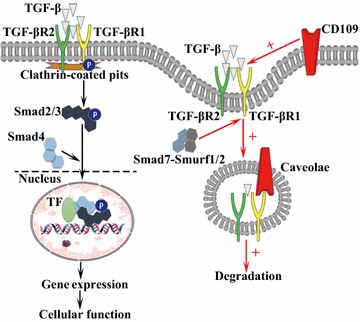
CD109 and TGF-β signaling. TGF-β binds the TGF-βR2, which recruits and phosphorylates the TGF-βR1. Activated TGF-βR1 propagates the signal downstream by directly phosphorylating Smad2 and Smad3. Phosphorylated R-SMADs then form heteromeric complexes with Smad4, combined with transcription factors, regulate gene transcription and cell function. TGF-β receptors internalize via the clathrin-coated pits or the caveolar pathway. CD109 increases TGF-β binding to TGF-β receptors and promotes TGF-β receptor localization to the caveolae, ultimately increases TGF-β receptor endocytosis and facilitates TGF-β receptor degradation. The inhibitory effects of CD109 require Smad7 expression and Smurf2 ubiquitin ligase activity

Besides, TGF-β1 is a potent inhibitor of growth in most epithelial cells [5]. Hagiwara et al. [17] demonstrated that oral SCC cell lines overexpression CD109 accelerated cell proliferation and impaired the anti-proliferative effect mediated by TGF-β1. In contrast, SCC cells with CD109 knockdown exhibited slower cell growth [17]. A high level of CD109 expression inhibited Smad2 phosphorylation, thus attenuated TGF-β1/Smad2 signaling and impairs TGF-β1-mediated suppression of cell growth, CD109 knockdown increased Smad2 phosphorylation by TGF-β1 stimulation [17]. Although CD109 also regulates Smad1/5 signaling [54], it has not been connected with the development of SCC. Together, CD109 facilitates the development of SCCs via inhibition of the TGF-β-Smad2/3 pathway (Fig. 2).

### CD109 and STAT3 signaling

Signal transducer and activator of transcription factor 3 (STAT3) is critical to cell proliferation, differentiation, migration, survival, and oncogenesis [37, 55]. Litvinov et al. [56] reported that the expression of CD109 protein was markedly decreased in psoriatic epidermis as compared to adjacent uninvolved skin. However, CD109 mRNA expression is unchanged in psoriatic plaques in comparison with normal skin, suggesting a possibility that CD109 protein release is enhanced in psoriatic keratinocytes [56]. They suggested that released/soluble CD109 is able to induce molecular changes that are known to occur in psoriasis [56]. In vitro, they found that transfection of CD109 siRNA down-regulates STAT3, release of CD109 from the cell surface of cultured human keratinocytes. In addition, exogenous/recombinant CD109 induces STAT3 signaling in human keratinocytes [56]. Besides, Chuang et al. reported that CD109 expression was dramatic upregulated in metastatic lung adenocarcinoma cells, and cells expressing a CD109 shRNA (shCD109) showed a dramatic reduction in STAT3 phosphorylation. STAT3 knockdown greatly reduced metastases, and restoration of STAT3 activity increased the ability of shCD109-expressing cells to metastasize [57]. Upon activation, STAT3 is phosphorylated by the non-receptor protein tyrosine kinases janus kinase 2 (JAK2), leading to the formation of STAT3 dimer and translocation into the nucleus [58, 59]. However, inhibition of JAK kinase activity in fibroblasts overexpressing CD109 reduced phosphorylated STAT3 to a level similar to that in the parental cells expressing low levels of CD109, suggesting that CD109-induced STAT3 phosphorylation requires JAK kinase activity. Thus, JAK/STAT3 signaling might mediate the effects of CD109 in tumor growth and metastasis [57].

Although knockdown of CD109 in human keratinocytes and lung adenocarcinoma cells downregulates STAT3 signaling in vitro [56, 57], the CD109-deficient mice displayed opposite results. Mii et al. [60] generated CD109-deficient mice, which exhibited skin abnormalities including epithelial hyperplasia, and inflammatory cell infiltration. They reported that STAT3 phosphorylation in CD109-deficient mice was significantly higher compared to the wild type mice. In addition, up-regulation of STAT3 signaling is associated with increased proliferation and impaired differentiation of keratinocytes [60].

The discrepancy of the results from in vitro and in vivo studies might be caused by the systematic changes of microenvironment in the tissues of CD109 deficient mice. Loss of CD109 in all the cells in mice might modify the subcutaneous microenvironment which activates STAT3 signaling in keratinocytes. In addition, CD109 may exert distinct regulatory effects in different cell types, leading to cell-type specific modification in STAT3 signaling. In addition to keratinocytes, CD109 is expressed in endothelial cells, epithelial cells, and fibroblasts [3, 5, 19, 42], which participate in constitute the skin tissue. However, to date the relationship of CD109 and STAT3 signaling have not been explored in these cell types.

### CD109 and EGFR signaling

**Fig. 3:**
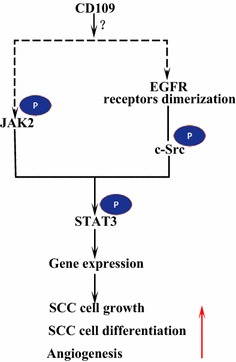
CD109 and EGFR/STAT3 signaling. CD109 facilitates EGFR signaling. The dimerized EGFR receptors phosphorylate c-Src, which in turn phosphorylates STAT3. CD109 may also activates STAT3 through phosphorylation of JAK2, then promote SCC cell growth, differentiation, and angiogenesis

Epidermal growth factor receptor (EGFR) is a member of the ErbB family of receptors. Upon ligand binding by EGF, EGFR forms dimers, either homodimers or heterodimers with another member of the ErbB family HER2 [61]. The dimerized receptors auto-phosphorylate each other and then phosphorylate the non-receptor protein tyrosine c-Src kinase, which activates STAT3 [58]. The activation of EGFR promotes cell migration, survival, and proliferation. In malignant tumors EGFR over-expression is correlated with the depth of invasion of the tumor and linked to poorer prognosis [61]. Mutations that lead to EGFR overexpression are detected in lung SCC [62], head and neck SCC [63], and esophagus SCC [64]. The membrane-anchored CD109 in SK-MG-1 cells directly interacts with EGFR and enhances EGF signaling, which subsequently increases cell migration and invasion, while the secreted CD109 has no effect on EGF signaling [65]. EGFR might mediate the effects of CD109 on STAT3 signaling, which requires further studies to elucidate (Fig. 3).

To date the broad picture of tumorigenesis and development of SCC remain incomplete. There are cross-talks between TGF-β and STAT3 or EGFR signaling pathways. CD109 is likely one of the key effectors of the signaling network regulating SCCs. However, no direct evidence has been reported to delineate the roles of CD109 in pathogenesis of SCCs.

### Perspectives

Studies from human tissue samples indicate that CD109 is highly expressed in SCCs of multiple organs, particularly in well-differentiated malignant squamous cells [15, 17]. By detection of CD109 expression with immunohistochemistry in human tissues, CD109 may potentially acts a biomarker to determine the progression of SCC. Current studies suggest that CD109 is highly expressed in well-differentiated SCCs and its expression is lower in un-differentiated SCCs [15, 17]. However, it is not clear whether CD109 is associated with vascular invasion, metastasis, and prognosis after surgery. Therefore, further studies are needed to explore the clinicopathological significance of CD109 in SCCs in a larger sample size.

CD109 is a GPI-linked glycoprotein, which enables it to be released from the membrane [56]. The soluble form of CD109 also affects the binding of TGF-β to its receptors, and subsequently modulate SCC progression [53, 56]. Litvinov et al. [56] found that CD109 released from the cell surface into the extracellular milieu, and the released form of CD109 retains its ability to induce intracellular signaling pathways. Besides, Sakakura et al. [66] reported that serum CD109 was released by xenografted tumor and it increases proportionally with the volume of tumor xenograft. Therefore, detection of serum CD109 level might help monitor tumors overexpressing CD109 including SCCs.

Exosomes communicate primary tumor lesion and its niche via its package containing selected proteins or other molecules [67]. The isolation and analyses of circulating tumor-associated exosome may serve as biomarkers for diagnosis of cancer patients. Exogenous CD109 has been identified as a component of exosome secreted from transfected 293 cells, making it a promising target for exosome diagnosis [66]. Still, to date CD109 have not been reported in exosomes derived from SCC or other tumor cells. The underlying mechanisms of CD109 packaging into exosomes deserve more detailed investigation.

In addition to the potential as a biomarker, CD109 might be a target for therapeutic approaches. CD109 is a membrane protein [3], which can be targeted directly by specific antibodies or enzymes. CD109 might also be recognized by targeted vehicles for drug delivery. However, the detailed roles of CD109 in pathogenesis of SCCs are still unclear. For example, the relationship of lower expression of CD109 and undifferentiated SCCs needs to be defined. CD109 intervention might only be considered in clinical practice after the risks and benefits are evaluated carefully.

## Conclusions

Squamous cell carcinoma (SCC) is one of the most common cancers of epithelial origin. CD109 is a cell-surface antigen and belongs to the α2-macroglobulin-C3, C4, C5 family of thioester-containing proteins. Studies from human tissue samples indicate that CD109 is highly expressed in SCCs of multiple organs, particularly in well-differentiated malignant squamous cells. Besides, CD109 is a co-receptor of TGF-β and negatively regulate TGF-β signaling via formation of a receptor complex with TGF-βR1 and TGF-βR2. In addition, CD109 interact with JAK/STAT3 and EGFR signaling pathways in other cell types. However, there is no direct evidence that CD109 regulate the development of SCCs by these signaling pathways. Further studies are needed to investigate the role of CD109 highly expression in pathogenesis of SCCs and the underlying molecular mechanisms.

## Abbreviations

AC: adenocarcinoma
ASC: adenosquamous carcinoma
CSCC: cutaneous squamous cell carcinoma
ESCC: esophagus squamous cell carcinomas
EGFR: epidermal growth factor receptor
EGF: epidermal growth factor
GBC: gallbladder cancer
GPI: glycosylphosphatidylinositol
GSCC: gallbladder squamous cell carcinoma
JAK2: janus kinase 2
LSCC: lung squamous cell carcinoma
NSCLC: non-small-cell lung cancer
NMSC: non-melanoma skin cancer
OSCC: oral squamous cell carcinoma
PSCC: penile squamous cell carcinoma
qRT-PCR: quantitative real time polymerase chain reaction
SARA: Smad anchor for receptor activation
SCC: squamous cell carcinoma
STAT3: signal transducer and activator of transcription factor 3
TGF-β: transforming growth factor-β
TMA: tissue microarrays
